# The Niche Awakens: Comprehension of Cancer Stem Cells’ Microenvironment to Plan New Therapeutic Strategies

**DOI:** 10.3390/cells15110997

**Published:** 2026-05-29

**Authors:** Dominika Pigoń-Zając, Maria Bryczek, Agata Leszczuk, Adrian Zając

**Affiliations:** 1 Department of Human Physiology of the Chair of Preclinical Sciences, Medical University of Lublin, Radziwiłłowska 11, 20-080 Lublin, Poland; dominika.pigon-zajac@umlub.edu.pl; 2 Department of Functional Anatomy and Cytobiology, Institute of Biological Sciences, Maria Curie-Sklodowska University, Akademicka 19, 20-033 Lublin, Poland; maria.bryczek.2000@gmail.com; 3 Institute of Agrophysics, Polish Academy of Sciences, Doświadczalna 4, 20-290 Lublin, Poland; a.leszczuk@ipan.lublin.pl

**Keywords:** cancer biology, cancer stem cells, niche, tumor microenvironment, metastasis

## Abstract

Cancer stem cells (CSCs) are a highly influential population of tumor cells involved in tumor initiation, progression, metastasis, recurrence, and resistance to therapy. Although CSCs have been widely investigated, their behavior cannot be understood solely through intrinsic cellular features, as these cells strongly depend on a specialized supportive microenvironment known as the CSC niche. In this review, we discuss the CSC niche as a dynamic and therapeutically relevant ecosystem that is distinct from, but closely connected with, the broader tumor microenvironment. Particular attention is given to stromal cells, immune cells, endothelial cells, extracellular matrix components, hypoxia, cytokines, chemokines, and metabolic stress as regulators of CSC self-renewal, plasticity, dormancy, immune escape, epithelial–mesenchymal transition, metastatic dissemination, and survival under therapeutic pressure. We further consider how CSC–niche interactions contribute to pre-metastatic niche formation and tumor relapse. Finally, we outline emerging therapeutic strategies aimed at disrupting CSC-supportive signals, including approaches targeting developmental pathways, angiogenesis, hypoxia, extracellular matrix remodeling, immunosuppressive networks, and cytokine-mediated communication. Overall, this review emphasizes that targeting the CSC-supportive microenvironment is essential for limiting metastasis, recurrence, and long-term treatment failure.

## 1. Introduction

Cancer diseases are still among the most significant challenges in modern medicine due to their complex biology and the profound impact on patients’ lives and healthcare systems. Often associated with the term “mutation,” cancer development is primarily explained by the mutational theory, which attributes tumorigenesis to the accumulation of genetic mutations that disrupt normal cellular growth control mechanisms. However, this perspective does not fully encapsulate the multifaceted nature of cancer. Beyond genetic mutations, tumor progression is significantly influenced by epigenetic modifications and the dynamic interactions within the tumor microenvironment (TME), collectively shaping tumor heterogeneity. One of the most promising and transformative discoveries in cancer biology has been the identification of cancer stem cells (CSCs). These cells exhibit remarkable plasticity and adaptability, having the ability to self-renew, differentiate, evade apoptosis, and initiate metastases. They exist within specialized regions of the tumor microenvironment known as niches. These niches support the growth, survival, and stem-like properties of CSCs by activating key signaling pathways, enabling CSCs to drive tumor initiation, progression, and dissemination to distant sites.

This study aims to elucidate the critical role of the niche in supporting CSCs and their malignant potential. By focusing on specific components of the niche, researchers and clinicians hope to develop therapeutic strategies that can disrupt tumor progression along with metastasis factors that remain the primary causes of cancer-related mortality.

### Cancer Diseases and Their Clinical Aspects

Cancer is characterized by the uncontrolled proliferation of abnormal cells, which form tissues with the ability to invade and destroy surrounding structures. These cells evade programmed cell death processes, such as apoptosis, and their unchecked growth results in suppressor genes and proto-oncogenes, disrupting the tightly regulated balance between cell proliferation and growth inhibition. As a result, proteins that stimulate cell division are triggered, while inhibitory mechanisms fail, leading to unregulated cell growth and tumor development. This chronic, genetically driven process often unfolds over many years, significantly impairing the quality of life of affected individuals [1].

Carcinogenesis typically progresses through multiple stages: initiation, promotion, progression, and metastasis. Each stage represents distinct biological and molecular changes that collectively lead to tumor development and dissemination. The prognosis of cancer largely depends on early detection. Oncological therapies are most effective when applied in the initial stages of the disease. However, the outlook for patients with advanced-stage cancer is still poor, even with optimized treatments. This challenge is clear in epidemiological data. For instance, in Poland alone, 171,200 new cases of cancer and 100,300 deaths were recorded in 2019, reflecting the persistent burden of this disease [2]. Screening programs designed for early detection, coupled with significant advances in medical research, have improved survival rates for certain cancers. However, recurrence remains a major obstacle, as many patients experience disease relapse even after undergoing successful initial treatment.

A central reason for therapeutic failure is the pervasive drug resistance of cancer cells, which underscores the urgent need for innovative treatment strategies. Current therapies predominantly target the bulk of the tumor, focusing on mature, rapidly dividing cells. While this approach may achieve partial remission, it often leaves the tumor’s source unaddressed. Consequently, the likelihood of recurrence and metastasis is still high [3]. The origin of the disease lies in a subpopulation of cells with distinct characteristics akin to pluripotent stem cells, known as cancer stem cells (CSCs) [4]. These cells play a pivotal role in the initiation and progression of tumors. Understanding CSC biology has become a major focus of cancer research, offering hope for the development of therapies that can target the root cause of the disease rather than merely addressing its symptoms.

In this review, we describe at the CSC niche not just as a region around cancer stem cells, but as an important feature of tumor biology that impacts how these cells behave. CSCs do not behave in isolation; their capacity to survive, stay plastic, defy immune control, resist treatment, and contribute to metastasis is heavily impacted by signals from their immediate surroundings. As a result, we examine the CSC niche in connection to the larger tumor microenvironment, with a focus on stromal, vascular, immunological, extracellular matrix, hypoxia-related, and cytokine- or chemokine-mediated interactions. We also discuss how recent advances in single-cell and spatial technologies, organoid models, and immuno-oncology have increased our knowledge of CSCs’ dynamic interactions with their niches. Finally, we discuss treatment options for disrupting this supporting network, including techniques that target developmental signaling pathways, hypoxia and angiogenesis, extracellular matrix remodeling, immunosuppressive processes, and cytokine/chemokine signaling. By combining the mentioned elements, the current study emphasizes that effective anti-CSC measures must attack not just CSCs but also the niche that allows them to persist, adapt, propagate, and reemerge following therapy [5,6,7,8,9,10,11].

## 2. Cancer Biology: Cancer Stem Cells

Cancer stem cells (CSCs) represent a specialized subpopulation within the heterogeneous tumor mass. These cells exhibit a unique set of characteristics that distinguish them from other tumor cells, including their ability to self-renew, initiate tumors, and resist therapeutic interventions. Even a small number of CSCs can drive tumor recurrence and regrowth, as shown by xenotransplantation studies. These experiments prove that cells isolated from primary tumors and transplanted into immunocompromised mice can initiate new tumors with remarkable efficiency [12]. CSCs are increasingly recognized as key players in therapeutic resistance. Understanding their biology and the molecular mechanisms that underlie their role in tumor progression is a critical area of research. By targeting CSCs, it may be possible to address the root causes of tumor development and recurrence, paving the way for more effective cancer therapies [13].

### 2.1. The Origin of Cancer Stem Cells

**Figure 1:**
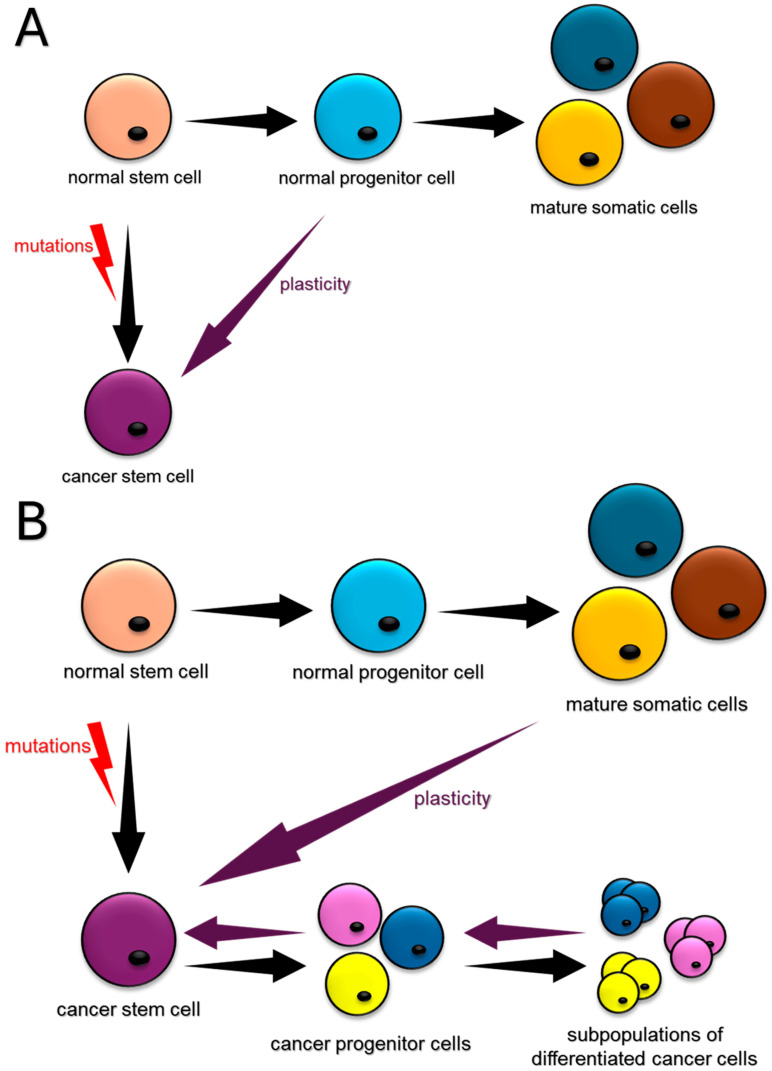
CSCs may arise from normal stem or progenitor cells after the accumulation of oncogenic alterations (**A**). Secondly, CSCs may also emerge from differentiated cancer cells that regain stem-like properties through cellular plasticity. Both mechanisms may contribute to tumor heterogeneity, therapy resistance, and relapse (**B**).

The origin of cancer stem cells (CSCs) remains an area of active investigation, with two dominant theories proposed in the literature (Figure 1).

The first suggests that CSCs arise from normal stem cells (SCs) or progenitor cells that acquire mutations. This theory is supported by the phenotypic similarities between CSCs and their normal counterparts, particularly their ability to self-renew and differentiate [14]. According to this hierarchical model, mutations transform normal stem cells into CSCs, which then drive tumorigenesis by generating the diverse cell types found within the tumor [4,13]. This process parallels the differentiation pathways of normal stem cells, from stem to progenitor to fully differentiated cells [4]. In this model, CSCs exhibit limited proliferation but maintain the ability to self-renew. Progenitor cells, on the other hand, divide rapidly to produce differentiated tumor cells. However, progenitor cells may also revert to a stem-like state through cellular plasticity, a process that complicates the hierarchical model. This plasticity enables dynamic transitions between cell states, contributing to tumor heterogeneity and complicating therapeutic targeting [13,15].

An alternative theory proposes that CSCs originate from mature somatic or differentiated tumor cells that regain stem-like properties. This process highlights the role of plasticity in tumor progression and suggests that CSCs can arise from multiple sources, contributing to their phenotypic diversity and adaptability. This adaptability presents a major challenge for therapies, as it enables CSCs to resist treatment and drive tumor recurrence and metastasis. In some cases, dormant CSCs may remain quiescent for extended periods, influenced by genetic, epigenetic, or microenvironmental factors. These dormant cells can later reinitiate tumor growth, underscoring the complexity of CSC biology and its implications for cancer therapy.

It is incorrect to see these two models as mutually exclusive. CSCs develop in various malignancies by a variety of processes, such as dedifferentiation of more mature tumor cells, transformation of normal stem or progenitor cells, or therapy-induced acquisition of stem-like characteristics. Each mechanism’s proportionate contribution most likely varies depending on the kind of tumor, the stage of the disease, and the therapeutic setting. Because CSCs reflect a dynamic and reversible cellular state rather than a fixed cell population, this poses a significant barrier for anti-CSC treatment [16,17,18].

### 2.2. Characterization of Cancer Stem Cells

Cancer stem cells (CSCs), often referred to as tumor-initiating cells (TICs), are a subpopulation within tumors that are responsible for their growth, recurrence, and metastasis. These cells have a unique set of properties that distinguish them from other tumor cells and make them particularly challenging to target. As the cornerstone of tumor heterogeneity, CSCs play a pivotal role in carcinogenesis, from their initiation to the establishment of distant metastases [19,20]. They form a dynamic and highly adaptive population that continuously interacts with the tumor microenvironment (TME), which in turn enhances their ability to survive, proliferate, and evade therapeutic pressures [21].

Cancer stem cells are characterized by several hallmark features, including self-renewal, dormancy, differentiation into diverse tumor cell types, and limited proliferation under normal conditions. They undergo two types of cell division: symmetric division (SD), which generates two identical CSCs, and asymmetric division (AD), which produces one cancer stem cell and one progenitor or differentiated cell. In normal stem cells, these processes are tightly regulated to maintain homeostasis. However, in CSCs, this balance is disrupted, with one mode of division often dominating, depending on the tumor type and stage of progression [15]. Asymmetric division contributes to tumor heterogeneity by creating diverse cell types while ensuring the persistence of the CSC pool. Symmetric division, on the other hand, prevents CSC depletion, maintaining their population over time. The ability of differentiated cells to revert to a stem-like state through plasticity further enhances the adaptability of CSCs, enabling them to withstand environmental and therapeutic challenges. In addition to their proliferative and regenerative capabilities, CSCs exhibit remarkable resistance to various forms of cellular stress, including DNA damage, oxidative stress, and hypoxia. They also demonstrate enhanced expression of molecular pathways associated with drug resistance, immune evasion, and metastatic potential [22,23]. This combination of traits makes CSCs a formidable adversary in the context of cancer therapy, as they contribute to the failure of standard treatments and drive disease recurrence.

### 2.3. Hypoxia and Angiogenesis

As cancer stem cells initiate tumor formation, the process of carcinogenesis progresses through its subsequent stages. During the promotion phase, the proliferation of cancer cells intensifies, leading to a rapid increase in tumor mass. In the initial stages of progression, tumors consist of approximately one million cells that are sufficiently nourished by nearby blood vessels, ensuring an adequate supply of oxygen, nutrients, and growth factors [24]. However, as the tumor grows larger and more complex, its expanding cell population begins to outpace the capacity of existing blood vessels to meet its metabolic demands. This spatial and metabolic imbalance results in hypoxia, a hallmark of all solid tumors, as regions of the tumor become deprived of oxygen [25].

In response to hypoxic conditions, rapidly proliferating cells produce hypoxia-inducible factor 1 (HIF-1), a heterodimeric protein composed of α and β subunits that mediates cellular adaptation to oxygen deprivation [26]. HIF-1 and its counterpart HIF-2 (hypoxia-inducible factor 2) play central roles in regulating the expression of vascular endothelial growth factor (VEGF), a potent pro-angiogenic factor. VEGF binds to specific receptors on endothelial cells, triggering the formation of new blood vessels. These newly formed vessels are often structurally and functionally abnormal, exhibiting tortuosity, dilation, and increased permeability [27]. Despite the induction of angiogenesis, tumors remain poorly oxygenated, perpetuating the cycle of hypoxia and adaptation [28]. Hypoxia has far-reaching effects on tumor biology, influencing critical processes such as cell proliferation, angiogenesis, and metastasis. Elevated hypoxia-inducible factor levels are associated with poor patient prognosis, as they enhance the invasiveness and therapeutic resistance of tumor cells [22,28]. CSCs are particularly well-suited to survive and thrive in hypoxic environments. They utilize adaptive mechanisms, such as metabolic reprogramming that favors glycolysis over oxidative phosphorylation, to maintain their malignant phenotype and resist treatment. Hypoxia also activates key signaling pathways, including WNT/β-catenin and TGF-β (transforming growth factor β), which further regulate CSC maintenance and stem-like properties [27,29,30].

Regions of extreme hypoxia within the tumor microenvironment serve as specialized niches for CSCs. These niches provide structural and biochemical support, promoting CSC survival and malignant behavior. The interplay between hypoxia, angiogenesis, and CSC biology underscores the critical role of the tumor microenvironment in shaping the progression and therapeutic resistance of cancer.

## 3. The Cancer Stem Cell Niche and Its Involvement in Tumor Progression

### 3.1. The Tumor Microenvironment

**Figure 2:**
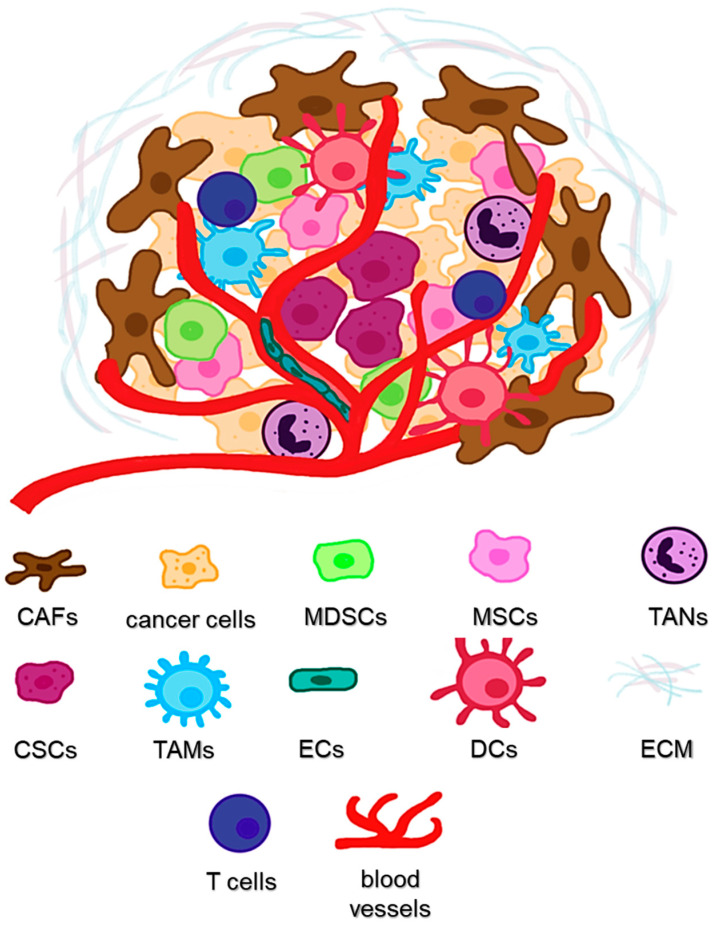
Main cellular and extracellular components of the tumor microenvironment. The tumor microenvironment contains cancer cells, CSCs, stromal cells, immune cells, endothelial cells, blood vessels, and extracellular matrix components. Mentioned elements interact with each other and support tumor growth, immune evasion, angiogenesis, invasion, and therapeutic resistance. CAFs—cancer-associated fibroblasts; MDSCs—myeloid-derived suppressor cells; MSCs—mesenchymal stem cells; TANs—tumor-associated neutrophils; CSCs—cancer stem cells; TAMs—tumor-associated macrophages; ECs—endothelial cells; DCs—dendritic cells; ECM—extracellular matrix.

A tumor consists not only of rapidly proliferating cancer cells but also of various surrounding components that form its immediate environment. This milieu, known as the tumor microenvironment (TME), is an extraordinarily complex and dynamic system composed of stromal cells, endothelial cells, infiltrating immune cells, the extracellular matrix (ECM), and soluble factors such as cytokines, hormones, and growth factors (Figure 2).

Together, these elements interact with the cancer cells, influencing their behavior and, in turn, being modified by them. The reciprocal interactions within the TME drive tumor heterogeneity, playing a crucial role in cancer progression and are correlated with drug-resistance development and patient survival outcomes. Cancer cells within the tumor microenvironment can manipulate their components to create conditions favorable for their survival and proliferation. This not only exacerbates tumor aggressiveness but also fosters resistance to conventional therapies. The diversity within the TME, including differences in cell type and molecular signaling, poses significant challenges for effective treatment. For instance, immune cells infiltrating the tumor can adopt either pro-tumorigenic or anti-tumorigenic roles depending on the signaling cues they receive from the microenvironment. Thus, the tumor microenvironment is not a passive bystander but an active participant in tumor evolution and progression [21].

### 3.2. Structure of the Cancer Stem Cell Niche and Its Functions

Within the tumor microenvironment, cancer stem cells occupy a specialized microenvironment called the niche. This niche is critical for maintaining the CSC population and preserving their unique stem-like properties, including self-renewal, plasticity, and resistance to therapy. Although distinct from the broader TME, the CSCs’ niche remains in constant interaction with other components of the tumor environment, influencing and being influenced by its surroundings [20,23]. CSCs actively recruit and remodel nearby cells to create a supportive niche. These recruited components include cancer-associated fibroblasts (CAFs), mesenchymal stem cells (MSCs), tumor endothelial cells (TECs), tumor-associated macrophages (TAMs), and myeloid-derived suppressor cells (MDSCs). Together, mentioned elements work to sustain CSCs’ characteristics, promote metastasis, and enhance angiogenesis and immune evasion [31,32]. For example, CAFs contribute to ECM remodeling, which facilitates CSCs’ migration, while TAMs and MDSCs suppress immune responses, creating a more permissive microenvironment for tumor progression.

The CSC niche is a functionally defined component of the larger tumor microenvironment rather than a completely distinct anatomical compartment. Many of its constituents, such as stromal cells, endothelial cells, immune cells, extracellular matrix, hypoxia, cytokines, and chemokines, are also present in the microenvironment that sustains non-CSC tumor cells. The result of these interactions is where the main distinction is found. Niche-derived signals sustain immune escape, metastatic competence, self-renewal, plasticity, dormancy, and treatment resistance in CSCs. Similar signals may primarily promote invasion, proliferation, survival, or metabolic adaptability in different tumor cells. For treatment, this distinction is crucial. When it comes to common TME components, focusing on the CSC niche might not be entirely selective. By concentrating on CSC-specific dependencies, such as developmental signaling, dormancy-related programs, CSC-associated receptor expression, immune-evasion pathways, or cytokine and chemokine axis that maintain stem-like states, more selectivity may be attained. Therefore, rather than being viewed as a distinct anatomical structure, the CSC niche should be viewed as a functional unit inside the larger TME. Tumor kinds and even different parts of the same tumor may have diverse compositions. While immunological, stromal, or extracellular matrix-related processes are more significant in certain malignancies, vascular and hypoxic signals may predominate in CSC maintenance in others. One of the primary causes of the continued difficulty in developing universal niche-targeted medicines is this context dependency [6].

Key developmental signaling pathways, such as WNT, Hedgehog, and Notch, are also active within the niche. These pathways, shared with normal stem cells, play a critical role in maintaining CSC properties and promoting tumor growth. For instance, MSCs within the niche produce interleukins like IL-6 and IL-8, activating the NF-κB (nuclear factor kappa-light-chain-enhancer of activated B cells) pathway, which enhances cancer stem cells’ survival and promotes tumor progression [29,30,31]. This dynamic interplay underscores the importance of the CSCs’ niche as both a driver of malignancy and a potential therapeutic target. The other mechanism that plays a crucial role in reinforcing CSC stemness and invasiveness is hypoxia. Hypoxia-inducible factors, activated under low oxygen conditions, regulate various signaling pathways, including TGF-β and TNF-α (tumor necrosis factor α), and trigger metabolic shifts favoring glycolysis over oxidative phosphorylation. This metabolic reprogramming supports cancer stem cells’ survival and resistance to therapies [27]. Additionally, hypoxia induces the secretion of CXCL12 (C-X-C motif chemokine 12) by CSCs, which recruits other stem cells into the niche and facilitates their acquisition of stem-like properties, further promoting tumor progression [27]. Despite growing knowledge about the cancer stem cell niche, therapeutic interventions targeting it remain challenging. The interactions within the niche not only sustain CSCs’ survival but also contribute to their proliferation, invasion, and resistance to treatments, highlighting the niche as a critical barrier to successful cancer therapy [10].

#### 3.2.1. Cancer-Associated Fibroblasts

Cancer-associated fibroblasts (CAFs) are among the most prominent stromal cells in the TME and play a vital role in shaping the CSC niche. Unlike normal fibroblasts, which maintain tissue homeostasis, CAFs are transformed by tumor-secreted factors such as fibroblast growth factor (FGF) and platelet-derived growth factor (PDGF). These factors induce a state of persistent activation in CAFs, rendering them resistant to apoptosis and promoting their tumor-supportive functions [12,33]. CSCs further activate CAFs through Hedgehog signaling, perpetuating a feedback loop that enhances tumor progression. Activated CAFs secrete factors like hepatocyte growth factor (HGF) and transforming growth factor-beta (TGF-β), which support CSC traits and promote differentiation. HGF, for example, drives dedifferentiation into CSCs via the WNT pathway, while TGF-β induces epithelial–mesenchymal transition (EMT), a process that enhances cell migration and invasion [15,29,30]. CAFs also release IL-6, which enhances CSC survival and proliferation, and matrix metalloproteinases (MMPs), which remodel the ECM to facilitate tumor cell invasion and metastasis [34]. CAFs are not a homogeneous population, though. Different CAF subsets promote immune suppression, ECM remodeling, tumor development, or, in certain situations, limit tumor progression. This variability restricts straightforward CAF-depletion tactics and encourages more focused methods meant to obstruct particular CAF-derived signals that sustain CSCs [35,36].

#### 3.2.2. Tumor Endothelial Cells

Endothelial cells (ECs) form the inner lining of blood vessels and are essential for vascular homeostasis, mediating processes like vasodilation and preventing excessive smooth muscle proliferation [37]. Under normal physiological conditions, ECs have a low proliferative capacity, with cell division occurring approximately once every 150 days in adults [38]. However, in the context of tumors, hypoxic conditions induce an “angiogenicswitch,” stimulating ECs to initiate the formation of new blood vessels, or neovascularization. CSCs secrete factors like VEGF (vascular endothelial growth factor) and CXCL12, which disrupt endothelial junctions and increase vascular permeability, enabling tumor cells to intravasate into the bloodstream. Migrating ECs, guided by these signals, form new blood vessels that are structurally abnormal, often exhibiting tortuous, dilated, and permeable characteristics [39]. These vessels not only supply nutrients and oxygen to the growing tumor but also facilitate the dissemination of CSCs to distant organs, thereby promoting metastasis. In the TME, tumor endothelial cells (TECs) acquire malignant characteristics. They secrete factors such as IL-6 and VEGF, which maintain CSC traits and drive metastasis through pathways like Notch signaling. Hypoxia-induced VEGF plays a vital role in angiogenesis, driving tumor progression and facilitating metastasis [15]. By enabling both nutrient supply and CSC dissemination, TECs are integral to the interplay between angiogenesis and tumor growth.

#### 3.2.3. Mesenchymal Stem Cells

Mesenchymal stem cells (MSCs) are multipotent progenitor cells capable of differentiating into various specialized cell types, including osteoblasts, adipocytes, and chondrocytes. Their unique ability to migrate to hypoxic tissues makes them highly relevant in tumor biology, as these characteristics align closely with the microenvironment of growing tumors. This migratory behavior allows MSCs to localize to tumor niches, where they play a pivotal role in stimulating angiogenesis, maintaining CSC niches, and providing immune protection to cancer cells.

In tumor microenvironments, CSCs secrete interleukin-6 (IL-6) to attract MSCs. Once recruited, MSCs amplify the malignant properties of CSCs by producing cytokines such as IL-6, IL-8, and CXCL12, which activate the NF-κB signaling pathway. This interaction promotes CSC proliferation, sustains their stem-like properties, and enhances their survival in a hostile microenvironment [31,40,41]. Additionally, MSCs produce Gremlin1, a protein that inhibits the activity of transforming growth factor-beta (TGF-β), thereby supporting CSC stemness. Simultaneously, MSCs secrete pro-angiogenic factors such as vascular endothelial growth factor (VEGF) and platelet-derived growth factor (PDGF), which facilitate the formation of abnormal but functional blood vessels to nourish the tumor. Moreover, MSCs contribute to epithelial-mesenchymal transition (EMT)—a process crucial for cancer cell migration and metastasis—via the secretion of tumor necrosis factor-alpha (TNF-α) and TGF-β. These secreted factors not only increase CSC invasiveness but also their metastatic potential. Furthermore, MSCs actively promote the establishment of immunosuppressive niches within tumors. They activate regulatory T cells and inhibit the activity of effector B and T lymphocytes, thus preventing effective immune surveillance. This immunomodulatory role of MSCs significantly enhances tumor progression, making them a critical component of the CSC niche [41,42].

Regarding MSCs, while they can promote tumor progression by suppressing anti-tumor immunity and differentiating into cancer-associated fibroblasts, a substantial body of evidence also supports their intrinsic anti-tumor effects [43,44]. For instance, MSCs have been shown to suppress tumor growth by downregulating the PI3K/AKT signaling pathway, inducing apoptosis through TRAIL, and inhibiting angiogenesis [45,46],. Notably, the anti-cancer properties appear to be particularly pronounced in MSCs derived from the umbilical cord (UC-MSCs), which exhibit tumor-inhibiting effects across various cancer types, including leukemia, breast cancer, and glioma, in contrast to the often tumor-promoting effects observed with bone marrow-derived MSCs [46]. In line with these findings, recent studies using both murine adipose-derived MSCs and human UC-MSCs in mouse tumor models have not observed any pro-tumorigenic effects, further supporting the safety and potential therapeutic efficacy of these specific MSC populations [47,48]. Furthermore, the inherent tumor-homing property of MSCs makes them ideal vehicles for targeted delivery of anti-cancer agents. In addition, MSCs can be engineered to deliver therapeutic proteins (e.g., IFNs, TNFSFs), oncolytic viruses, or be loaded with chemotherapeutic drugs such as paclitaxel, serving as “Trojan horses” to concentrate the anti-tumor payload directly within the tumor microenvironment while minimizing systemic toxicity [46,47,48,49].

#### 3.2.4. Extracellular Matrix

The extracellular matrix (ECM) is a dynamic and highly organized network of macromolecules, including collagen, elastin, glycoproteins, and proteoglycans. In healthy tissues, the ECM provides structural support and regulates various cellular processes such as adhesion, migration, proliferation, and differentiation. However, in the context of cancer, the ECM becomes dysregulated, promoting tumor progression, metastasis, and resistance to therapies [50,51]. The ECM interacts closely with CSCs, maintaining their stem-like properties and supporting their survival within the niche. Integrins, a family of transmembrane receptors, play a pivotal role in anchoring CSCs to the ECM and activating intracellular signaling pathways essential for migration, invasion, and proliferation. Increased ECM stiffness enhances integrin binding, further promoting CSC migration, invasion, and resistance to chemotherapeutic agents. Specific ECM components contribute directly to CSC maintenance and tumor progression. For example, Tenascin-C (TNC) supports CSC proliferation and migration through activation of NOTCH and WNT signaling pathways. Galectin-1 (Gal-1), another ECM component, promotes immune evasion by inducing apoptosis in T-cells, thereby helping tumors escape immune surveillance [29,52,53,54]. Osteopontin (OPN) binds to integrins and CD44, enhancing CSC radioresistance and metastatic potential, while periostin (POSTN) facilitates tumor growth and immune evasion. ECM remodeling, driven by matrix metalloproteinases (MMPs), further supports tumor progression. MMPs degrade structural ECM components like collagen, releasing embedded cytokines such as TGF-β. This promotes EMT, enhancing CSC invasiveness and enabling their escape from the primary tumor. Altered ECM architecture also impacts immune cell infiltration, differentiation, and activation, fostering a microenvironment conducive to tumor growth and CSC maintenance [30,51,55].

#### 3.2.5. Immunosuppressive Cells

The immune system is a key player in detecting and eliminating abnormal cells, including those undergoing malignant transformation. In the initial stages of carcinogenesis, immune cells effectively suppress tumor development. However, as the disease progresses, cancer cells evolve mechanisms to evade immune surveillance. This process, termed immunoediting, involves reprogramming immune cells to support, rather than inhibit, tumor progression. The result is the establishment of an immunosuppressive microenvironment that tolerates tumor growth and shields cancer cells from immune attacks. Cancer stem cells are integral to this process, as they recruit and modulate various immune cell populations, including myeloid-derived suppressor cells (MDSCs), tumor-associated macrophages (TAMs), tumor-associated neutrophils (TANs), cytotoxic T lymphocytes (CTLs), and natural killer (NK) cells. By orchestrating these interactions, CSCs create a pro-tumorigenic and immunosuppressive niche that facilitates their survival, proliferation, and metastasis [32,56].

#### 3.2.6. Tumor-Associated Macrophages

**Figure 3:**
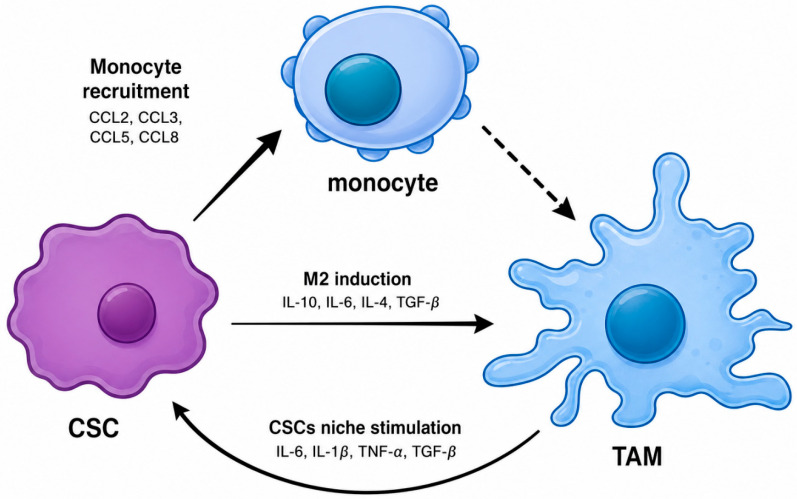
CSC-mediated regulation of tumor-associated macrophages. CSCs secrete chemotactic factors, including CCL2, CCL3, CCL5, and CCL8, which recruit monocytes into the tumor microenvironment. CSC-derived cytokines, such as IL-4, IL-6, IL-10, and TGF-β, promote macrophage polarization toward an M2-like TAM phenotype. In turn, TAMs release IL-1β, IL-6, TNF-α, and TGF-β, which support CSC maintenance, niche formation, inflammation, and tumor progression.

Tumor-associated macrophages (TAMs) are among the most abundant immune cells in the tumor microenvironment and can adopt pro- or anti-tumor functions depending on their polarization. M1 macrophages are classically activated and exhibit anti-tumor properties, including the ability to phagocytose cancer cells and release pro-inflammatory cytokines. Conversely, M2 macrophages are alternatively activated, displaying immunosuppressive properties that support angiogenesis, tissue remodeling, and tumor progression. A low M1/M2 ratio is often correlated with poor clinical outcomes in cancer patients [57]. CSCs actively recruit macrophages and polarize them toward the M2 phenotype by secreting chemokines such as CCL2 and cytokines like IL-6, which activate the STAT3 signaling pathway. This reprogramming suppresses anti-tumor cytokines, enabling TAMs to support CSC stemness and survival. TAMs also promote EMT and protect CSCs from microenvironmental stresses, enhancing their metastatic capabilities. Key factors secreted by TAMs include IL-6, TGF-β, and TNF-α, all of which play critical roles in CSC maintenance and tumor progression (Figure 3) [58,59].

#### 3.2.7. Myeloid-Derived Suppressor Cells

Myeloid-derived suppressor cells (MDSCs) are a highly heterogeneous population of immature immune cells originating from the bone marrow. These cells are critically involved in tumor-mediated immune suppression, particularly by targeting T-cell activity and thereby limiting the efficacy of immunotherapy approaches [59]. MDSCs exhibit their immunosuppressive effects through the production of reactive oxygen species (ROS) and arginine metabolism, which inhibits the activation and proliferation of T-cells. Additionally, they promote the development of regulatory T-cells (Tregs), further weakening the immune response against tumors. Beyond immune modulation, MDSCs actively contribute to tumor progression by stimulating angiogenesis and metastasis. They achieve this by secreting various pro-tumorigenic factors, including prostaglandin E2 (PGE2), interleukin-6 (IL-6), and nitric oxide (NO), which collectively enhance CSC stemness and metastatic potential. For instance, in cancers such as cervical, ovarian, and breast cancer, these molecules help CSCs maintain their stem-like characteristics and invade distant tissues. CSCs play a reciprocal role by recruiting MDSCs to the tumor site, leveraging their immunosuppressive properties to create a microenvironment conducive to tumor growth and metastasis. The recruitment and expansion of MDSCs are driven by a range of factors secreted by tumor cells, including granulocyte colony-stimulating factor (G-CSF), IL-6, and vascular endothelial growth factor (VEGF) [60]. These interactions not only sustain the immunosuppressive milieu but also enhance the survival and propagation of CSCs, underscoring the critical role of MDSCs in cancer progression.

#### 3.2.8. Tumor-Associated Neutrophils (TANs)

Tumor-associated neutrophils (TANs) are an abundant and versatile immune cell population within the tumor microenvironment. While neutrophils typically play a protective role against infections and tissue injury, in cancer, their functional phenotype can be reprogrammed to support tumor progression. TANs can polarize into two distinct phenotypes: the anti-tumorigenic N1 type, which promotes inflammation and immune responses, and the pro-tumorigenic N2 type, which enhances tumor growth, angiogenesis, and metastasis. The N2 phenotype dominates in most cancers, driven by tumor-secreted factors such as G-CSF and VEGF [61]. Cancer stem cells (CSCs) influence TAN polarization through signaling molecules like transforming growth factor-beta (TGF-β), which directs neutrophils toward the N2 phenotype. This polarization enhances CSC-like traits in tumor cells, such as invasiveness and the ability to metastasize. However, under certain conditions, N2 TANs can revert to their N1 phenotype, potentially regaining their anti-tumor functions [59]. TANs also collaborate with mesenchymal stem cells (MSCs) to further tumor progression. Through the secretion of chemokines such as CCL17 and CCL22, TANs recruit regulatory T-cells to the tumor site, suppressing anti-tumor immunity and enabling CSCs to thrive. This interplay demonstrates the critical role of TANs in establishing an immunosuppressive environment that facilitates tumor progression [62].

## 4. The Influence of the Niche on the Migratory Potential of Cancer Stem Cells

### 4.1. Metastasis Process Overview

Metastasis is a complex and multi-step biological process that represents the most lethal aspect of cancer. It begins with the escape of cancer cells from the primary tumor, followed by invasion into the extracellular matrix (ECM), intravasation into the bloodstream, survival in circulation, extravasation into distant tissues, and finally, colonization and growth in secondary organs. Despite its complexity, metastasis is highly inefficient, with fewer than 1% of cancer cells surviving the journey to establish metastatic sites. Nevertheless, metastasis accounts for most cancer-related deaths, highlighting its clinical significance [63]. A critical subset of cancer cells, known as metastatic cancer stem cells (MCSCs), possesses unique properties that enable them to complete this process. These cells exhibit tumor-initiating capacity, stress resistance, and phenotypic heterogeneity, enabling them to adapt to the challenging conditions encountered during metastasis [64].

**Figure 4:**
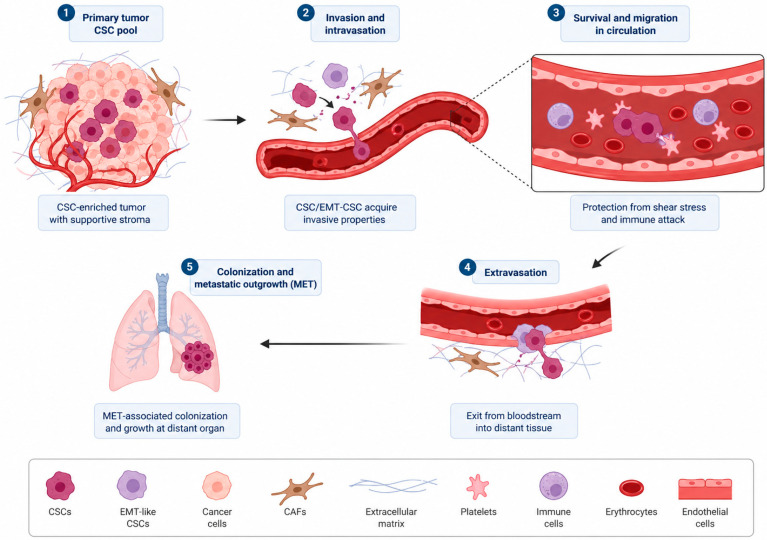
Role of CSCs in the metastatic cascade. CSCs and EMT-like CSCs can detach from the primary tumor, invade the surrounding extracellular matrix, enter the bloodstream, survive immune and mechanical stress in circulation, adhere to endothelial cells, extravasate into distant tissues, and initiate metastatic colonization. CAFs—cancer-associated fibroblasts; EMT—epithelial-to-mesenchymal transition; MET—mesenchymal-to-epithelial transition. Own elaboration based on [65] generated with BioRender.com.

MCSCs acquire migratory capabilities through epithelial-to-mesenchymal transition (EMT), a process that enables their detachment from the primary tumor and invasion into surrounding tissues (Figure 4).

Conversely, mesenchymal-to-epithelial transition (MET) facilitates colonization and proliferation in secondary sites. Both EMT and MET are regulated by signals derived from the CSC niche and ECM components, which play a crucial role in determining the metastatic potential of MCSCs [12]. Research suggests that circulating tumor cells (CTCs) and disseminated tumor cells (DTCs)—both of which are often enriched with CSCs—adhere to the “seed and soil” hypothesis. According to the mentioned theory, metastatic success depends not only on the intrinsic properties of the “seed” (MCSCs) but also on the suitability of the “soil” (the pre-metastatic niche) [42,66,67]. This intricate interplay between CSCs and their niche underscores the need for targeted therapies that disrupt the metastatic process at multiple stages, potentially improving outcomes for patients with advanced cancers.

### 4.2. Epithelial-to-Mesenchymal Transition: Key to Initiating Metastasis

Epithelial-to-mesenchymal transition (EMT) is a critical mechanism that enables cancer stem cells (CSCs) to detach from the primary tumor, invade surrounding tissues, and acquire motility. This transformation involves significant morphological and molecular changes, including the downregulation of epithelial markers such as E-cadherin and the upregulation of mesenchymal markers like N-cadherin, vimentin, and matrix metalloproteinases (MMPs). These changes collectively enhance extracellular matrix (ECM) degradation and boost CSC invasiveness, facilitating metastasis [68].

It is incorrect to think of EMT as a straightforward transition between mesenchymal and epithelial states. Hybrid epithelial-mesenchymal phenotypes, which combine high adaptability, survival ability, and metastatic competence, are acquired by many tumor cells. Because they enable tumor cells to adjust to therapeutic pressure and local niche cues, these intermediate states may be particularly significant for CSC biology. When developing anti-metastatic or anti-CSC methods, it is important to keep in mind that the significance of EMT differs according to the kind of tumor [69,70].

The EMT process is triggered by various tumor microenvironment signals. Transforming growth factor-beta (TGF-β) secreted by tumor-associated macrophages (TAMs) and MMPs released by cancer-associated fibroblasts (CAFs) are pivotal in driving EMT. TGF-β modulates gene expression, while MMPs cleave E-cadherin and degrade the ECM, creating physical pathways for CSC migration [55]. Additionally, hypoxia and chronic inflammation—hallmarks of the tumor microenvironment—further stimulate EMT-related pathways, solidifying the transition. Interestingly, EMT has been intricately linked to the acquisition of stem-like properties in cancers such as breast, pancreatic, and colorectal cancer. EMT-induced cells often exhibit enhanced sphere formation, a hallmark of CSC populations, indicating their elevated tumorigenic potential [68].

### 4.3. Migration and Extravasation of Cancer Stem Cells

Circulating cancer stem cells (CTCs) face a highly hostile environment, with the immune system posing a constant threat. Approximately 0.2% of CTCs survive this phase, highlighting the challenges of metastasis. However, CSCs employ several mechanisms to enhance their survival. Reduced immunogenicity, secretion of immunosuppressive molecules, and adhesion to platelets are central strategies. Platelets form a physical barrier around CSCs, protecting them from immune attacks, particularly from natural killer (NK) cells. Additionally, platelet-derived TGF-β suppresses NK cell activity and enhances CSC invasiveness [65,71,72]. CTCs thrive in the oxygen-deprived conditions of the bloodstream due to their robust DNA repair mechanisms and ability to form hypoxic spheroids. These compact aggregates serve as “micro-niches,” providing protection from oxidative stress and supporting survival during circulation. For successful extravasation, CTCs adhere to endothelial cells through integrins or form thrombi that lodge in small vessels. CSC spheroids play a pivotal role in these processes, further enhancing metastatic potential. Upon extravasation, mesenchymal-to-epithelial transition (MET) enables CSCs to colonize secondary sites, transitioning from dormancy to active proliferation and forming secondary tumors [65,73].

### 4.4. Pre-Metastatic Niche and Colonization

The successful colonization of distant organs by disseminated CSCs depends on the establishment of a pre-metastatic niche—an environment conducive to their adaptation, proliferation, and reactivation from dormancy. These niches are formed through intricate interactions involving CSC niches, the tumor microenvironment, and bone marrow-derived cells (BMDCs). Tumor-derived secreted factors (TDSFs) such as TNF-α, TGF-β, VEGF, and hypoxia-inducible factors (HIFs) play a significant role in recruiting BMDCs to shape the pre-metastatic site [74,75]. BMDCs are instrumental in remodeling the vasculature and releasing inflammatory cytokines, creating a favorable microenvironment for metastasis. For instance, VEGF and placental growth factor (PIGF) mobilize BMDCs to accumulate in fibronectin-rich lung niches, preparing these sites for metastatic colonization [74]. Additionally, tumor-derived extracellular vesicles (EVs), including exosomes, transport proteins, lipids, and nucleic acids such as miRNA to distant sites. These EVs modulate the local microenvironment by promoting angiogenesis, immune suppression, and ECM remodeling [76]. Cytokines and chemokines secreted by the primary tumor also play a significant role in recruiting immunosuppressive cells such as TAMs, TANs, and MDSCs to the pre-metastatic niche. These cells suppress anti-tumor immune responses, creating conditions conducive to CSC colonization. For example, MDSCs release reactive oxygen species that inhibit T-cell activity, effectively enhancing the niche’s readiness for metastatic cells [77]. Pre-metastatic niches mirror the primary tumor microenvironment, allowing CSCs to replicate their initial niche conditions. Stromal cells, including TAMs and CAFs, support CSC traits through paracrine loops that sustain stemness and invasiveness. Simultaneously, ECM breakdown by metalloproteinases releases pro-metastatic factors like TGF-β and VEGF, further promoting tumor progression and metastasis [31].

## 5. CSCs and Their Niche as a Therapeutic Targets

**Figure 5:**
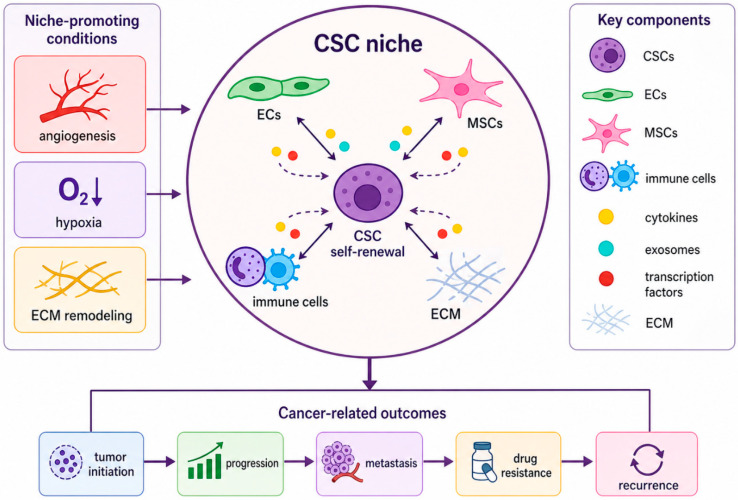
Impact of CSC–niche interactions on cancer progression and therapeutic resistance. CSC-supportive niche conditions, including angiogenesis, hypoxia, and extracellular matrix remodeling, help maintain CSC self-renewal and survival. Interactions with endothelial cells, mesenchymal stem cells, immune cells, cytokines, extracellular vesicles, transcription factors, and ECM components contribute to tumor initiation, progression, metastasis, drug resistance, and recurrence. CSCs—cancer stem cells; ECs—endothelial cells; MSCs—mesenchymal stem cells; ECM—extracellular matrix. Own elaboration based on [78] generated with BioRender.com.

The discovery of cancer stem cells (CSCs) and their involvement in all cancer stages (Figure 5) has opened new avenues for therapeutic development, aiming for complete cancer remission. However, targeting CSCs presents challenges due to their inherent traits, such as drug resistance, immune evasion, dormancy, and phenotypic plasticity. Moreover, targeting CSCs alone may not suffice, as their surrounding niche significantly contributes to their malignancy.

Therapeutic efforts for the CSC niche should not be limited to eradicating CSCs directly. The niche delivers signals that enable CSCs to survive, remain latent, adapt to stress, avoid immune control, and resume tumor development following treatment. As a result, niche-directed treatment may boost the efficacy of traditional chemotherapy, radiation, targeted therapy, and immunotherapy [5,16,79,80].

One significant technique is to block the developmental signaling pathways that keep CSC stemness. The WNT, Notch, and Hedgehog pathways govern CSC self-renewal, differentiation, EMT, and treatment resistance. Their suppression may lower the number of CSCs and hinder tumor regrowth. However, these pathways also control normal stem cells, increasing the danger of toxicity. This restricts the use of single-agent pathway inhibitors while encouraging their usage in carefully planned combinations [16,81,82].

Another crucial tactic focuses on angiogenesis and hypoxia. Through HIF-dependent signaling, metabolic adaptability, VEGF production, and treatment resistance, hypoxic conditions improve CSC survival. While extensive artery pruning can exacerbate hypoxia and encourage a more aggressive CSC phenotype, anti-angiogenic treatment may reduce vascular support for CSCs. This implies that methods that interfere with hypoxia-induced plasticity and survival processes should be used with anti-angiogenic therapy [83,84,85].

Another important therapeutic target is the extracellular matrix. CSC migration, invasion, and drug resistance may be supported by increased matrix stiffness, changed collagen organization, integrin signaling, and matrix metalloproteinase activity. Treatments that disrupt integrin-mediated adhesion, MMP activity, or ECM remodeling may reduce the capacity of CSCs to invade and spread. Because CAFs play a significant role in ECM deposition, remodeling, and cytokine generation, these strategies should be taken into consideration in conjunction with treatments that target CAFs [86,87,88].

A further option for therapy is the immunosuppressive CSC niche. TAMs, MDSCs, TANs, regulatory T cells, and fatigued cytotoxic T cells all interact with CSCs. These interactions reduce the effectiveness of immunotherapy and shield CSCs from immune clearance. This immune protection may be weakened by targeting IL-6, IL-8, CXCL12/CXCR4, TGF-β, CCL2/CCR2, and other cytokine or chemokine axis. These tactics enhance reactions to cancer vaccines, adoptive cell treatment, and immune checkpoint inhibitors [5,7,89].

Furthermore, the pre-metastatic niche should be considered seriously whereas designing therapeutics. Through affecting the extracellular matrix, attracting immunosuppressive cells, and promoting angiogenesis, CSCs and tumor-derived extracellular vesicles may activate distant organs for metastatic invasion. Regression and diffusion may be prevented by focusing on these early niche-forming mechanisms. In general, combination strategies targeting CSCs, their niche, and interactions between them will probably be necessary for effective anti-CSC treatment. Compared to treatments that just target the tumor mass, this strategy may be more successful in reducing recurrence, metastatic spread, and long-term treatment failure [90,91,92,93].

Therapeutic strategies disrupting niche–CSC interactions hold promise in overcoming these challenges [12]. Key developmental signaling pathways such as WNT, Notch, and Hedgehog are essential for CSC maintenance and represent primary therapeutic targets. Inhibiting the Notch pathway reduces CSC populations in cancers like breast, colon, and medulloblastoma, while combination therapies with chemotherapy prevent metastasis and relapse [30,94]. Hedgehog signaling inhibitors, such as cyclopamine, suppress pancreatic cancer CSCs, limiting tumor growth and invasion [94]. Anti-angiogenic strategies, such as the VEGF inhibitor bevacizumab combined with chemotherapy, improve outcomes in cancers like breast, lung, and colorectal. By depleting CSC niches through reduced angiogenesis, these therapies hinder tumor progression. However, the resilience of CSCs under hypoxia necessitates the development of alternative approaches targeting compensatory pathways [95,96]. Hypoxia-induced factors (HIFs) play a pivotal role in CSC survival and metastasis under oxygen-deprived conditions. Small-molecule inhibitors targeting HIFs or associated pathways like PI3K-AKT-mTOR have demonstrated potential to reduce CSC malignancy and therapy resistance [97]. Targeting immunosuppressive cytokines such as IL-6, CXCL12, and IL-8 reprograms the CSC niche, making tumors more responsive to treatments. Reparixin, for instance, targets IL-8, reducing CSC-like phenotypes in vitro [32]. Similarly, ECM-targeting therapies, like MMP inhibitors, disrupt the structural integrity of the niche, curbing angiogenesis and metastasis [32].

Future therapeutic strategies integrating cancer stem cells-targeted and niche-targeted approaches (Table 1) hold the potential to revolutionize cancer treatment, enhancing patient outcomes by addressing the root of tumor resilience and progression.

This summary demonstrates that a single universal target is insufficient for CSC niche-targeted treatment. Tumor kind, illness stage, treatment history, and immunological environment all influence the niche’s heterogeneity and adaptability. Therefore, disruption of stromal, vascular, immunological, extracellular matrix, and cytokine-mediated support should be combined with CSC-intrinsic targeting in future therapeutic approaches. Recurrence, metastasis, and long-term treatment failure may be more effectively prevented using this strategy.

## 6. Summary

Cancer remains one of the most common and dangerous diseases today, posing a significant societal challenge. Despite advances in prevention, evolving diagnostic techniques, and continuous progress in medicine, statistical data on incidence and patient survival remain unsatisfactory. The mortality rate continues to be high, primarily due to disease recurrence, which can manifest even years after remission, and metastases to distant tissues, responsible for over 90% of cancer-related deaths. Traditional cancer therapies target mature, rapidly proliferating cells. However, this approach often fails to effectively identify and eliminate the root cause of the cancer. Furthermore, there is a considerable risk that some cells may survive treatment and lead to disease relapse in the future.

A groundbreaking discovery enabling a better understanding of tumor etiopathogenesis and the mechanisms underlying its progression is the identification of cancer stem cells (CSCs). The pivotal role of CSCs at every stage of carcinogenesis makes them a promising therapeutic target, with their complete elimination offering hope for overcoming primary tumors and preventing recurrence. However, while targeting this unique population is an attractive strategy, it does not guarantee complete success. The malignant phenotype of these cells results largely from their interactions with their supportive niche, which drives their growth and development while also creating favorable conditions for dormant, treatment-resistant populations. CSCs and their niche are the focus of intense research. Potential therapeutic models primarily aim to disrupt signaling pathways maintaining the stem-like phenotype, interfere with angiogenesis, modulate the immunosuppressive microenvironment, or inhibit cytokines produced by niche components. The diversity and dynamics of the microenvironment pose a significant challenge to fully understanding its mechanisms. Nevertheless, anti-cancer therapies targeting CSCs and their niches offer the possibility of less invasive and more selective treatments, providing hope for better clinical outcomes in oncology patients.

There are still a lot of important challenges to be addressed, though. Characterizing CSCs as a stable population is difficult since many stem-like traits can appear or disappear in response to treatment or niche signals. Additionally, the utility of universal therapeutic techniques is diminished by the fact that various tumor types have distinct CSC habitats. Future studies should thus focus on tumor-specific CSC–niche interactions, therapeutically relevant models, and combination therapies that target both CSCs and the microenvironmental support required for their survival and post-treatment reemergence.

## Figures and Tables

**Table 1:** Therapeutic targets and strategies directed against CSCs and their niche.

Target or Niche Component	Biological Role in CSC Maintenance	Representative Strategy	Expected Therapeutic Effect	Evidence Level	Main Limitations	References
WNT, Notch, Hedgehog pathways	Maintain CSC self-renewal, differentiation state, epithelial-to-mesenchymal transition, and therapy resistance	Pathway inhibitors used alone or with chemotherapy, radiotherapy, targeted therapy, or immunotherapy	Reduced CSC frequency, impaired tumor regrowth, reduced EMT and resistance	Preclinical and early clinical, tumor-type dependent	Toxicity due to effects on normal stem cells, pathway redundancy, resistance	[82]
Hypoxia and HIF signaling	Support CSC survival, dormancy, metabolic adaptation, VEGF production, immune escape, and resistance	HIF inhibitors, metabolic targeting, hypoxia-modifying strategies	Reduced hypoxia-driven plasticity and survival under therapy	Mainly preclinical, with some translational evidence	Tumor hypoxia is heterogeneous, systemic effects, limited biomarker guidance	[83]
Angiogenesis and vascular niche	Provide vascular support for CSCs and regulate oxygen and nutrient supply	Anti-VEGF or anti-angiogenic therapy combined with anti-hypoxia strategies	Reduced vascular support and CSC niche activity	Clinically used in selected tumors, CSC-specific effects mostly indirect	Excessive vessel pruning may worsen hypoxia and enrich CSC-like phenotypes	[98]
Extracellular matrix remodeling	Supports CSC adhesion, invasion, migration, metastasis, and drug resistance	Integrin inhibitors, MMP modulation, ECM remodeling inhibitors	Reduced invasion, metastatic spread, and therapy resistance	Mostly preclinical and translational	ECM complexity, compensatory remodeling, toxicity of broad MMP inhibition	[86]
Cancer-associated fibroblasts	Produce ECM, cytokines, chemokines, and growth factors that support CSC survival and invasion	CAF reprogramming or depletion, blockade of CAF-derived signals	Weakened stromal support for CSCs and improved therapy response	Preclinical and early translational	CAF heterogeneity, risk of removing tumor-restraining CAF subsets	[35]
TAMs, MDSCs, TANs, Tregs, exhausted T cells	Build an immunosuppressive CSC niche and reduce immune clearance	TAM or MDSC targeting, Treg modulation, immune checkpoint blockade combinations	Improved anti-tumor immunity and reduced CSC immune escape	Preclinical, translational, and clinical depending on strategy	Immune toxicity, tumor-specific immune context, adaptive resistance	[99]
IL-6, IL-8, CXCL12/CXCR4, TGF-beta, CCL2/CCR2 axes	Promote CSC survival, stemness, immune evasion, migration, and niche recruitment	Cytokine or chemokine blockade, receptor antagonists, combination immunotherapy	Disrupted CSC-niche communication and improved treatment response	Preclinical and early clinical depending on axis	Redundant signaling, systemic immune effects, patient selection needed	[5]
Pre-metastatic niche and extracellular vesicles	Prepare distant organs for CSC colonization through ECM remodeling, angiogenesis, and immune suppression	EV-targeting approaches, inhibition of niche-forming cytokines, anti-metastatic combinations	Reduced dissemination, metastatic colonization, and relapse	Mainly preclinical and emerging translational evidence	Lack of validated clinical inhibitors, difficulty monitoring early niche formation	[100]

## Data Availability

No new data were generated or analyzed in this study. All data discussed are available in the referenced publications.
